# Meta-analysis and pharmacoeconomic study of rasagiline versus selegiline in the treatment of Parkinson’s disease

**DOI:** 10.1080/07853890.2026.2711475

**Published:** 2026-08-19

**Authors:** Hanzong Zhou, Dandan Li, Junxian Yu, Zheng Gu, Hongtao Wei

**Affiliations:** ^a^ Beijing Friendship Hospital, Capital Medical University, Beijing, China; ^b^ Capital Medical University, Beijing, China; ^c^ University of Leeds, Leeds, UK

**Keywords:** Parkinson’s disease, rasagiline, selegiline, efficacy, pharmacoeconomics

## Abstract

**Objective:**

Given the persistent absence of direct head-to-head trials, this study aimed to evaluate the comparative efficacy, safety, and cost-effectiveness of rasagiline versus selegiline as early-stage monotherapy for Parkinson’s disease (PD), informing clinical selection and healthcare policies in China.

**Methods:**

A systematic search of PubMed, Embase, and the Cochrane Library identified randomized controlled trials (RCTs) up to April 2026. Focusing on short-term outcomes (10–16 weeks), an adjusted indirect treatment comparison (ITC) using placebo as a common anchor evaluated symptom improvement (UPDRS total scores) and adverse event (AE) incidence. For economic evaluation, a 2-year Markov model was constructed from a Chinese healthcare-system perspective. The incremental cost-effectiveness ratio (ICER) was calculated alongside robust sensitivity analyses.

**Results:**

Ten RCTs (rasagiline: 6; selegiline: 4) were included. The ITC revealed no statistically significant differences between rasagiline and selegiline in short-term symptomatic relief (Mean Difference = −0.82, 95% CI [−2.08, 0.44], *p* = 0.203) or AE risk (Odds Ratio = 0.83, 95% CI [0.50, 1.38], *p* = 0.475). The overall evidence certainty was rated as moderate. Economically, the base-case simulation indicated rasagiline yielded a marginal benefit of 0.0088 QALYs over selegiline but incurred an additional 17,111.10 Yuan. This resulted in an ICER of 1,951,505.55 Yuan/QALY, substantially exceeding the conventional willingness-to-pay threshold.

**Conclusion:**

Supported by moderate-certainty evidence, rasagiline and selegiline provide comparable short-term efficacy and safety for early-stage PD monotherapy. However, at its current pricing, rasagiline is not cost-effective. Significant price reductions or definitive proof of long-term superiority are required to justify its economic value.

## 1. Background and objective

Previous epidemiological studies have shown that the incidence and prevalence of Parkinson’s disease (PD) in China are 24.3/100,000 and 245.7/100,000, respectively, which are higher than the global average [1]. The all-cause mortality rate of PD patients is 2.22 times higher than that of the general population [2,3]. Treatment and care costs contribute significantly to the economic burden on both the healthcare system and individual patients.

### 1.1. Efficacy and safety

While previous meta-analyses [4–8] have established both rasagiline and selegiline as efficacious treatments for Parkinson’s disease, their findings are primarily derived from indirect comparisons. To date, direct head-to-head randomized controlled trials (RCTs) comparing these two agents remain conspicuously absent, and existing systematic reviews have yet to specifically address their short-term efficacy in early-stage monotherapy. Consequently, current clinical guidelines rely on evidence that necessitates strict transitivity assumptions, which may not fully capture the nuances of real-world practice. This study aims to bridge these gaps by providing a rigorous synthesis of the available data, specifically focusing on the initial phase of treatment.

### 1.2. Economic research

According to a disease burden survey involving 200 Chinese patients, the annual per capita economic burden for PD patients is 18,851.37 Yuan, accounting for 38.50% of the average annual income. Monoamine oxidase inhibitors (MAO-B) can improve motor symptoms of PD [9]. They also have certain disease-modifying effects [10]. Commonly used drugs include Selegiline and Rasagiline, with Rasagiline having stronger evidence from clinical studies for the management of motor complications[11]. This study compares the differences in drug efficacy, safety, and economic outcomes between Selegiline and Rasagiline through head-to-head meta-analysis and cost-utility analysis. Different from existing NMAs that focus solely on clinical outcomes, this study provides incremental value by integrating an indirect meta-analysis with a cost-utility analysis specifically tailored to the Chinese healthcare context. We aim to generate hypotheses regarding the comparative efficacy, safety, and economic outcomes of Selegiline and Rasagiline to guide future large-scale trials and reimbursement decisions in China.

## 2. Methods

### 2.1. Efficacy and safety

#### 2.1.1. Literature search method

A comprehensive systematic literature search was conducted across PubMed, Embase, and the Cochrane Library from database inception up to April 2026. The search strategy was constructed using a combination of Medical Subject Headings (MeSH) and free-text words, structured around three core domains combined with the Boolean operator ‘AND’.

For the first domain regarding the target population, the search terms included ‘Parkinson Disease’ [MeSH], ‘Parkinson*’, and ‘PD’. For the second domain encompassing the interventions and comparators, we utilized ‘rasagiline’ [Supplementary Concept/MeSH], ‘Azilect’, ‘selegiline’ [MeSH], ‘deprenyl’, ‘Eldepryl’, and ‘Zelapar’. To restrict the results to relevant study designs, the third domain applied the validated Cochrane Highly Sensitive Search Strategy for identifying randomized trials. This filter included the publication types ‘randomized controlled trial’ and ‘controlled clinical trial’, along with the MeSH term ‘clinical trials as topic’ [NoExp], and the text words ‘randomized’, ‘placebo’, ‘randomly’, and ‘trial’. No language restrictions were applied during the search process.

#### 2.1.2. Inclusion and exclusion criteria

Study eligibility was determined strictly based on the PICOS framework. The target population comprised adult patients with confirmed idiopathic Parkinson’s disease strictly undergoing early-stage monotherapy. We excluded patients with secondary parkinsonism, Parkinson-plus syndromes, severe cognitive impairment, or major psychiatric disorders. The intervention of interest was rasagiline monotherapy limited to the standard recommended dose of 1 mg/day or selegiline at a standard dose of 10 mg/day; studies using rasagiline in combination therapies were excluded. The comparator was either selegiline at a standard dose of 10 mg/day or a placebo. For outcomes, the primary endpoint was the short-term efficacy, mean change from baseline in the Unified Parkinson’s Disease Rating Scale (UPDRS) total score (10–16 Weeks). Secondary endpoints encompassed the mean change in UPDRS Part II and Part III scores, as well as the incidence of adverse events (AEs). Eligible study designs were restricted to English-language randomized controlled trials (RCTs). Observational studies, reviews, case reports, and animal studies were excluded.

#### 2.1.3. Study selection

The selection of studies was conducted in a two-stage screening process by two independent reviewers, following the inclusion and exclusion criteria (Zhou, Li).

First, the titles and abstracts of all records retrieved from the literature search were screened independently by the two reviewers to identify potentially relevant articles and exclude those that were clearly ineligible.

Next, the full texts of the remaining articles were retrieved and assessed independently by the same two reviewers against the pre-specified inclusion and exclusion criteria. Any disagreements or conflicts at either screening stage were resolved through discussion and consensus. If an agreement could not be reached, a third, senior reviewer (Wei) was consulted for adjudication and a final decision. We separately analyzed the efficacy and safety outcomes reported in the included studies

#### 2.1.4. Data extraction and conversion

Two investigators (Zhou and Li) independently extracted data from the included studies using a standardized, pre-piloted data extraction form developed in Microsoft Excel. In instances where the required short-term efficacy data were only presented graphically (e.g. line charts tracking UPDRS changes over specific early time points) and numerical values were not explicitly reported in the text or tables, data extraction was conducted using the WebPlotDigitizer software (version 4.8, https://automeris.io/WebPlotDigitizer/). Any minor discrepancies in the visually extracted values between the reviewers were resolved by calculating the average of their respective estimations or through discussion with a third reviewer to ensure optimal precision. If an agreement could not be reached, a third reviewer (Wei) was consulted for final adjudication.

Based on the predefined form, the following data were systematically extracted from each eligible study:

Study and Participant Characteristics: First author, disease staging, sample size for both experimental and control groups (E/C), and average age of participants (E/C).
Intervention Details: Specific treatments for the experimental and control groups, along with the specific timepoints for outcome assessment (follow-up duration).
Efficacy Outcomes (Continuous Variables): Short-term UPDRS total scores, specifically including the mean and standard deviation (mean ± SD) for UPDRS Part II, UPDRS Part III, and UPDRS total scores.
Safety Outcomes (Dichotomous Variables): The number of reported adverse events (AEs).
Risk of Bias Information: Data required for assessing the methodological quality across domains of the risk of bias tool.
To preserve data granularity and address the heterogeneity of clinical outcomes, we categorized the extracted data based on their types and processed them accordingly:
Continuous Variables (Efficacy): For efficacy endpoints relying on clinical symptom scores (i.e. UPDRS total, Part II, and Part III), we extracted the mean and SD at both baseline and follow-up. Where necessary, these values were used to calculate the mean change from baseline and its corresponding SD. We strictly avoided converting these continuous measures into binary outcomes to prevent any loss of statistical information.
Dichotomous Variables (Safety): Safety endpoints were analyzed as dichotomous data. We directly extracted the event counts of AEs, alongside the total sample size for each treatment arm, to evaluate the safety profiles of the interventions.

#### 2.1.5. Risk of bias assessment

The methodological quality and risk of bias for each included randomized controlled trial were assessed using the Cochrane Risk-of-Bias tool for randomized trials, version 2 (RoB 2). Two reviewers (Zhou, Li) independently evaluated each study, extracting relevant information from the full-text articles. Any discrepancies in the assessment were resolved through discussion and consensus. If an agreement could not be reached, a third senior reviewer (Wei) was consulted. The risk of bias for the included studies was presented using a Risk of Bias (RoB) summary plot.

To evaluate the risk of bias arising from selective outcome reporting, we specifically assessed ‘Bias in selection of the reported result’. This was done by comparing the outcomes pre-specified in study protocols or trial registries with those reported in the final publications.

Furthermore, at the synthesis level, we planned to assess the risk of bias due to missing results (e.g. publication bias) by constructing funnel plots and performing Egger’s test, provided that at least 10 studies were included in the meta-analysis. This assessment also informed the ‘Publication Bias’ domain within our GRADE evaluation for the certainty of evidence.

#### 2.1.6. Data processing method

Direct pairwise meta-analyses were initially performed using Review Manager (RevMan) version 5.4.1. Subsequently, to evaluate the comparative efficacy and safety of rasagiline versus selegiline, where direct head-to-head trials were lacking, an indirect treatment comparison (ITC) was conducted using Python version 3.13.7. Statistical significance was defined as a two-tailed *p* < 0.05.

To preserve the continuous nature of clinical data and avoid information loss, effect measures were selected strictly based on the data type:

Continuous Outcomes: As all included studies assessed clinical symptoms using the same measurement tool (the UPDRS scale), the Mean Difference (MD) with 95% confidence intervals (CIs) was utilized as the summary statistic, rather than the Standardized Mean Difference (SMD).
Dichotomous Outcomes: For safety endpoints, specifically the incidence of adverse events (AEs), the Odds Ratio (OR) with 95% CIs was calculated.

Statistical heterogeneity across the included studies was evaluated using the Chi-square test and the I^2^ statistic. A fixed-effects model was applied if heterogeneity was low (*p* > 0.10 or I^2^ < 50%). Conversely, if substantial heterogeneity was detected (*p* ≤ 0.10 or I^2^ ≥ 50%), a random-effects model was adopted. For the indirect comparisons executed in Python, the common comparator (e.g. placebo) was used as a bridge to calculate the indirect MDs and ORs. Critically, in cases of considerable statistical heterogeneity or conflicting results between subgroups, the overall pooled estimates were interpreted with caution or deliberately de-emphasized to preclude misleading clinical conclusions.

#### 2.1.7. Subgroup analysis

Subgroup analyses were predefined to explore potential sources of clinical or methodological variance, such as variations in follow-up duration or specific dosages. However, these analyses were planned strictly contingent upon the detection of substantial statistical heterogeneity (*p* > 0.10 or I^2^ < 50%). In the absence of significant heterogeneity, the pre-planned subgroup analyses were waived, as the overall pooled effect was considered highly consistent and reliable.

#### 2.1.8. Sensitivity analysis

To evaluate the robustness of the pooled results and further explore potential sources of substantial heterogeneity, a pre-planned sensitivity analysis was conducted. Specifically, we employed the leave-one-out approach, systematically omitting one included study at a time and recalculating the pooled effect estimates. This procedure allowed us to ascertain whether the overall conclusions were disproportionately skewed or driven by any single study, thereby validating the stability of our findings.

#### 2.1.9. Certainty of evidence

The certainty of the body of evidence for each pre-specified outcome was assessed using the Grading of Recommendations, Assessment, Development and Evaluations (GRADE) approach. The certainty of evidence was rated as high, moderate, low, or very low. Evidence from randomized controlled trials (RCTs) started at high certainty, while evidence from observational studies started at low certainty. We considered five domains for downgrading the certainty of evidence: risk of bias, inconsistency, indirectness, imprecision, and publication bias. For evidence from observational studies, we also considered three domains for upgrading: large magnitude of effect, dose-response gradient, and the effect of plausible residual confounding. The final certainty rating for each outcome was determined by two reviewers independently (Zhou, Li), with any disagreements resolved by discussion or consultation with a third reviewer (Wei).

### 2.2. Economic research

This economic evaluation was conducted and reported in accordance with the CHEERS 2022 reporting guidance [12].

#### 2.2.1. Perspective and methodology of economic research

Economic analysis was conducted by reviewing literature to collect data on the drug costs, treatment effects, and transition probabilities of using Selegiline and Rasagiline in PD patients, with the study adopting Chinese healthcare system perspective and calculating the incremental cost-effectiveness ratio (ICER).

#### 2.2.2. Model setting

A decision tree and Markov model were constructed using Treeage Pro 2022, assuming that all patients were in H-Y stage 1 at the start of the simulation, with an average age of onset for PD around 60 years, leading to a simulation duration of 2 years.

The sample size for the simulation was set to 1,000 individuals.

Discount Rate: According to guidelines [13], a 5% annual discount rate was applied to both costs and effects.

A China-specific study estimated a cost-effectiveness threshold of approximately 1.45 times GDP per capita, with estimated lower and upper bounds of 1.16 and 2.90 times GDP per capita, respectively [14]. In the base-case analysis, three times GDP per capita was adopted as the willingness-to-pay threshold.

Specific criteria are as follows: (1) If the Incremental Cost-Effectiveness Ratio (ICER) is less than one times the per capita GDP, the intervention is considered fully cost-effective; (2) If the ICER is between one and three times the per capita GDP, it is considered cost-effective and acceptable; (3) If the ICER exceeds three times the per capita GDP, the intervention is deemed economically infeasible.

#### 2.2.3. Outcome indicators

In our base-case economic model, baseline clinical characteristics and initial health states were parameterized based on a large international validation study [15] of the Hoehn and Yahr (H&Y) scale. The natural disease progression of Parkinson’s disease was simulated using 3-month transition probabilities between H&Y stages; these transition probabilities were derived from a comprehensive model [16], which synthesized progression data from 10 longitudinal studies comprising 3,318 patients. Baseline all-cause mortality for the 60–64 age group was obtained from the China 2020 Population Census Yearbook [17]. Survival was modeled by applying standard age-specific mortality rates which were further adjusted using PD-specific mortality multipliers. This annual rate was subsequently converted into the 3-month transition probability required for the model using the formula d3m = (1-qannual)1/4.

Regarding efficacy assessment, MDS-UPDRS scores were converted into UPDRS scores using a validated conversion equation to ensure consistency across the included trials according to the reported article [18]. It is noteworthy that because both MAO-B inhibitors were evaluated in the context of early monotherapy, their therapeutic efficacy was exclusively applied to early-to-mid-stage PD in our model. Cost calculation: The cost is obtained by adding direct and indirect costs. Direct costs are the price of drugs from each manufacturer multiplied by the patient’s dosage; indirect costs include ADR-related costs, administrative fees, transportation fees, caregiving fees, etc. All costs were adjusted to 2024 Chinese Yuan (YUAN) using the consumer price index. Since accurate data on lost work time and caregiving costs could not be obtained, only ADR-related costs were included in the indirect costs.

The Hoehn - Yahr staging system was used to determine the PD staging criteria, with H-Y stages 1-2.5 representing the early stages of the disease and H-Y stages 3–5 representing the middle and late stages [19,20].

QALY was calculated as an outcome indicator using H-Y staging and UPDRS. The utility values were calculated using the mapping algorithm: EQ-5D = 79.272 - 0.775 * UPDRS II - 0.008 * UPDRS III² [21].

Transition probabilities: Literature was reviewed to obtain the disease transition probabilities associated with Rasagiline and Selegiline interventions.

#### 2.2.4. Sensitivity analysis

The uncertainty of the model is calculated using univariate sensitivity analysis and probabilistic sensitivity analysis.

## 3. Results

### 3.1. Evaluation of effectiveness and safety

#### 3.1.1. Literature search results

**Figure 1.**
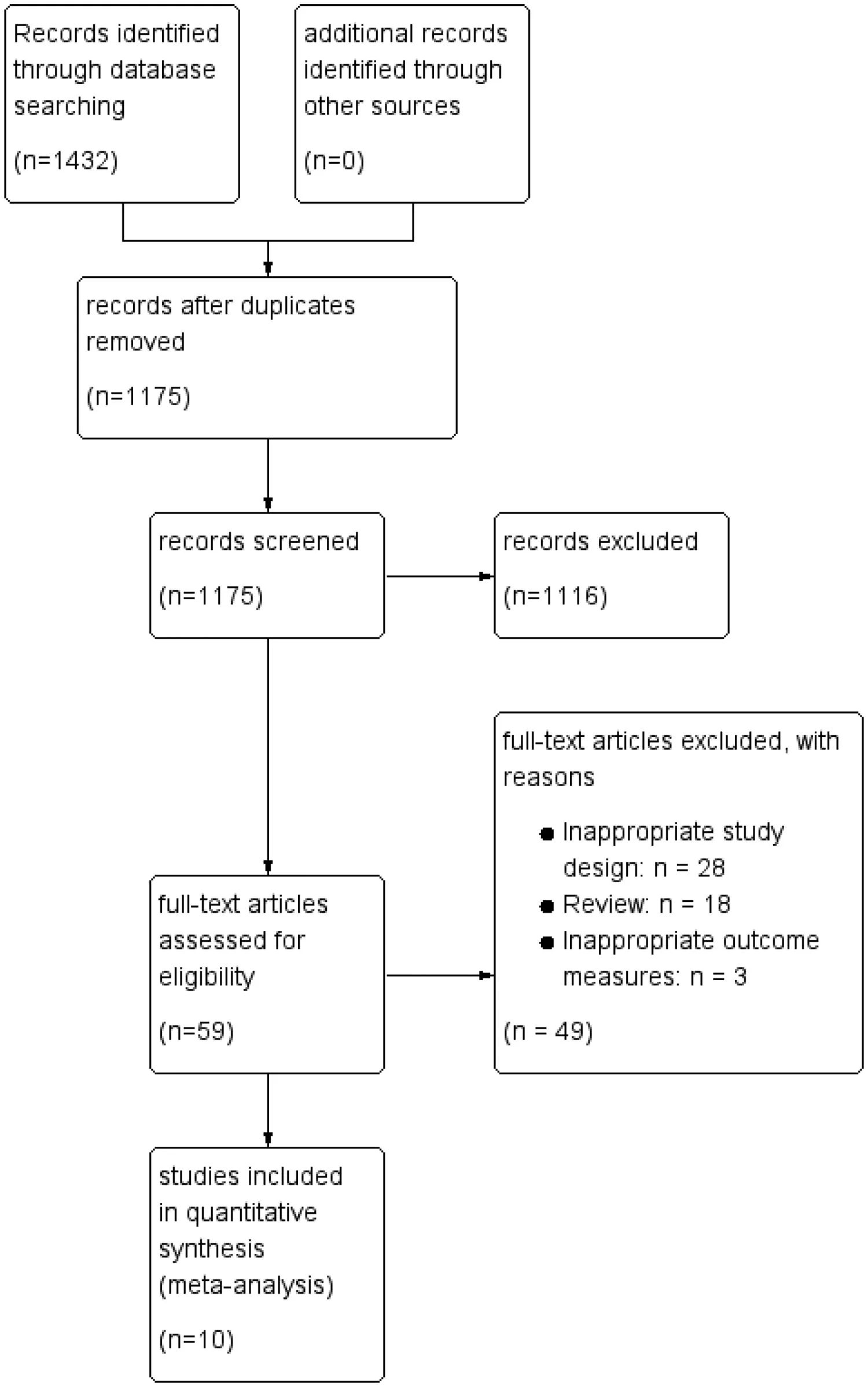
PRISMA flow diagram of study selection. The flowchart illustrates the identification, screening, eligibility, and inclusion process of the studies. n represents the number of records or articles.

A total of 1175 articles were retrieved, and ultimately 10 RCTs [22–31] (6 of rasagiline and 4 of selegiline) were included. 9 of them reported the main outcome. The Prisma [32,33] literature screening is shown in Figure 1.

The basic characteristics of the included studies are shown in Table 1.

**Table 1.** Characteristics of the included studies.

Author	Staging	Experimental Group	Control Group	Sample Size (E/C)	Average Age (E/C)	Timepoint	Outcome Measures	Adverse Events (n)	Serious Adverse Events (n)
Fabrizio Stocchi et al. [22]	H-*Y* ≤ 3	Rasagiline 1 mg/d	Placebo	204/204	63.3 (10.0)/62.9 (10.2)	16 Weeks	UPDRS II+III	106/106	NA
Matthew B. Stern et al. [23]	H-*Y* ≤ 3	Rasagiline 1 mg/d	Placebo	15/13	59.3 (8.6)/64.8 (9.4)	10 Weeks	UPDRS total	9/8	0/0
C. Warren Olanow et al. [24]	H-*Y* ≤ 3	Rasagiline 1 mg/d	Placebo	288/300	62.4 (9.7)/61.9 (9.7)	12 Weeks	UPDRS total	NA	NA
Parkinson Study Group [25]	H-*Y* ≤ 3	Rasagiline 1 mg/d	Placebo	134/138	61.6 (10.3)/60.5 (10.8)	14 Weeks	UPDRS total	109/110	6/4
Nobutaka Hattori et al. [26]	H-*Y* ≤ 3	Rasagiline 1 mg/d	Placebo	118/126	67.4 (9.0)/65.4(8.8)	14 Weeks	UPDRS II; UPDRS III	73/66	4/8
Zhenxin Zhang et al. [27]	H-*Y* ≤ 3	Rasagiline 1 mg/d	Placebo	65/65	58.5 (8.7)/59.5 (9.2)	14 Weeks	UPDRS total	27/30	0/4
S. Pålhagen et al. [28]	H-*Y* ≤ 3	Selegiline 10 mg/d	Placebo	81/76	63.3 (9.1)/64.2 (6.6)	12 Weeks	UPDRS total; II; III	NA	NA
Yoshikuni Mizuno et al. [29]	H-*Y* ≤ 3	Selegiline 10 mg/d	Placebo	146/146	63.9 (8.5)/64.2 (7.8)	12 Weeks	UPDRS II; UPDRS III	100/90	4/0
H. Allain et al. [30]	H-*Y* < 2.5	Selegiline 10 mg/d	Placebo	48/45	64.5 (8.5)/65.4 (10.1)	12 Weeks	UPDRS total; II; III	(11/47)/(9/42)	1/4
Parkinson Study Group [31]	H-*Y* ≤ 2	Selegiline 10 mg/d	Placebo	202/199	NA	12 Weeks	UPDRS total; II; III	NA	NA
Fabrizio Stocchi et al. [22]	NA	NA	28.6 (11.6)/28.5 (12.5)	NA	NA	3.28 (1.11)/2.94 (1.10)	NA	NA	NA
Matthew B. Stern et al. [23]	NA	NA	18.2 (6.5)/17.7 (7.9)	NA	NA	−1.8 (5.03)/−0.5 (2.03)	NA	NA	NA
C. Warren Olanow et al. [24]	NA	NA	20.6 (8.4)/20.2 (8.8)	NA	NA	−1.69 (5.43)/0.41 (4.68)	NA	NA	NA
Parkinson Study Group [25]	NA	NA	24.7 (11.3)/24.5 (11.6)	NA	NA	−1.67 (12.15)/0.98 (13.04)	NA	NA	NA
Nobutaka Hattori et al. [26]	7.2 (5.5)/7.0 (4.6)	27.2 (13.8)/26.8 (11.6)	NA	−0.47 (3.10)/1.07 (3.10)	−4.93 (6.37)/−2.27 (5.90)	NA	NA	NA	NA
Zhenxin Zhang et al. [27]	NA	NA	26.14 (1.5)/25.56 (1.9)	NA	NA	−2.96 (7.01)/−0.62 (7.01)	NA	NA	NA
S. Pålhagen et al. [28]	NA	NA	23.6 (11.1)/20.6 (10.9)	−0.3 (1.9)/0.3 (2.3)	−1.1 (4.3)/0.4 (4.0)	−1.7 (5.4)/1.0 (5.3)	NA	NA	NA
Yoshikuni Mizuno et al. [29]	6.00 (3.37)/6.20 ± 3.95	19.69 (8.24)/19.75 (8.50)	NA	−1.13 (3.56)/−0.29 (4.25)	−4.86 (8.92)/−2.69 (9.49)	NA	NA	NA	NA
H. Allain et al. [30]	8.1 (4.3)/8.4 (4.0)	22.5 (10.5)/20.2 (9.0)	32.7 (14.0) /30.3 (11.9)	−3.1 (3.80)/−2.3 (3.61)	−7.0 (11.09)/−3.7 ± 9.31	−11.3 (13.95)/−5.6 (12.95)	NA	NA	NA
Parkinson Study Group [31]	NA	NA	NA	−0.36 (2.57)/0.68 (2.69)	−0.93 (5.49)/0.76 (4.89)	−1.56 (7.04)/1.34 (6.70)	NA	NA	NA

#### 3.1.2. Meta-analysis results

##### 3.1.2.1. Efficacy Analysis Results

**Figure 2.**
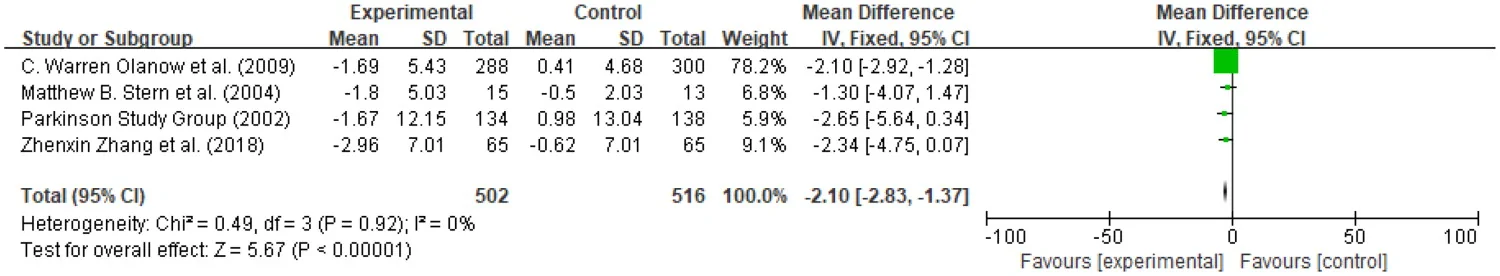
Forest plot of short-term clinical symptom improvement (UPDRS total score) for rasagiline versus placebo. The green squares represent the point estimate (mean difference) for each individual study, with the size of the square proportional to the study’s weight. The horizontal lines represent the 95% confidence intervals (CIs). The black diamond at the bottom represents the overall pooled effect size from the direct pairwise meta-analysis. SD: Standard Deviation; IV: Inverse Variance; CI: Confidence Interval; df: degrees of freedom.

**Figure 3.**
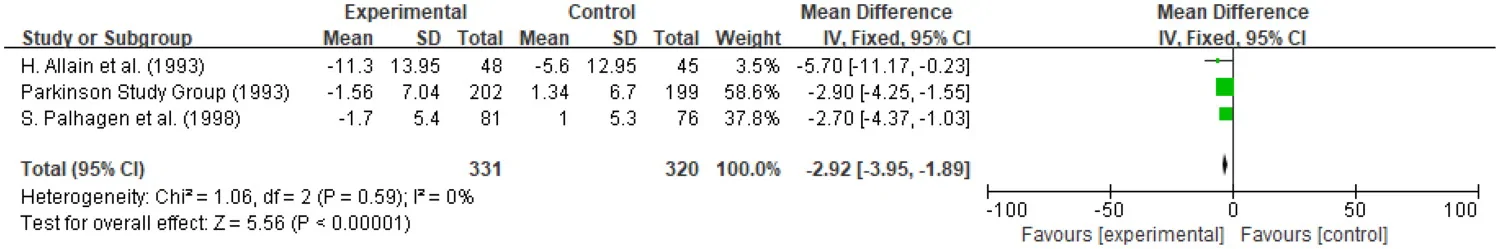
Forest plot of short-term clinical symptom improvement (UPDRS total score) for selegiline versus placebo. The green squares represent the point estimate (mean difference) for each individual study, with the size of the square proportional to the study’s weight. The horizontal lines represent the 95% confidence intervals (CIs). The black diamond at the bottom represents the overall pooled effect size from the direct pairwise meta-analysis. SD: Standard Deviation; IV: Inverse Variance; CI: Confidence Interval; df: degrees of freedom.

**Figure 4.**
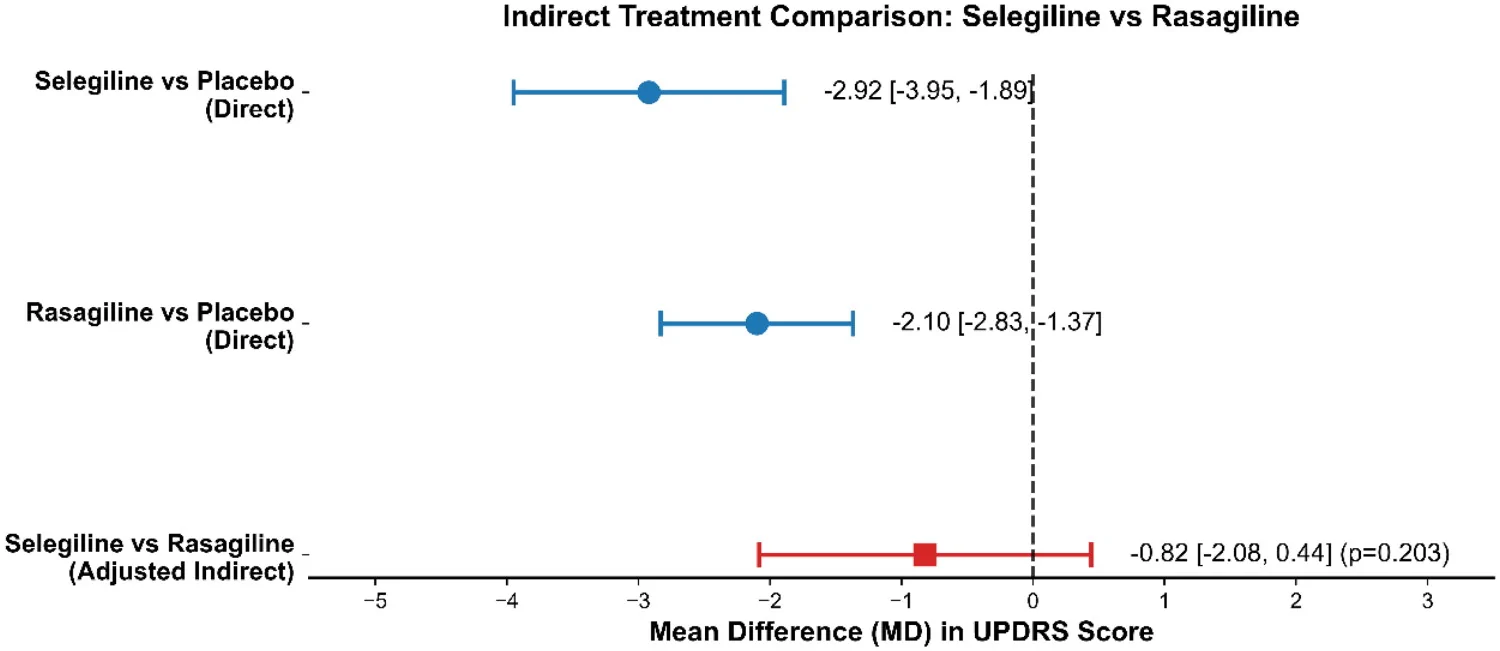
Adjusted indirect treatment comparison (ITC) of symptom improvement (UPDRS total score) between selegiline and rasagiline. The blue circles represent the pooled mean differences from the direct pairwise meta-analyses of selegiline or rasagiline versus placebo. The red square indicates the derived estimate from the Bucher indirect comparison of selegiline versus rasagiline. Horizontal lines denote the 95% confidence intervals (CIs). The vertical dashed line at zero represents the line of no effect. MD: Mean Difference; UPDRS: Unified Parkinson’s Disease Rating Scale; df: degrees of freedom.

Short-term Symptom Improvement (Indirect Treatment Comparison): Direct pairwise meta-analyses demonstrated that both selegiline and rasagiline significantly reduced UPDRS total scores compared to placebo (MD = −2.92, 95% CI [−3.95, −1.89], *p* < 0.00001; and MD = −2.10, 95% CI [−2.83, −1.37], *p* < 0.00001, respectively). However, the subsequent adjusted indirect treatment comparison utilizing the Bucher method [34] revealed no statistically significant difference in short-term efficacy between selegiline and rasagiline (MD = −0.82, 95% CI [−2.08, 0.44], *p* = 0.203). These findings suggest that both MAO-B inhibitors provide comparable short-term symptomatic relief for early-stage Parkinson’s disease. See Figures 2–4.

Two retrospective [35,36] observational studies comparing selegiline and rasagiline in patients with Parkinson’s disease demonstrate comparable clinical outcomes. In a real-world cohort of treatment-naive patients, there was no significant difference between the two medications regarding the time to initiation of levodopa therapy. Similarly, a 3-year case-control study reported no intra-class differences between selegiline and rasagiline in the clinical progression of motor and non-motor symptoms. Furthermore, both monoamine oxidase type B inhibitors exhibited equal efficacy in reducing daily levodopa requirements, lowering dyskinesia scores, and controlling overall motor symptoms.

##### 3.1.2.2. Safety analysis results

Direct pairwise meta-analyses were conducted to evaluate the incidence of adverse events (AEs) for both MAO-B inhibitors relative to control groups. The pooled results demonstrated that neither intervention significantly increased the risk of AEs compared to placebo. Specifically, the analysis of five trials for rasagiline yielded an Odds Ratio (OR) of 1.09 (95% CI [0.85, 1.40], *p* = 0.50), with absolute statistical homogeneity (I^2^ = 0%, *p* = 0.70). Similarly, the pooled data from two selegiline trials showed an OR of 1.31 (95% CI [0.84, 2.02], *p* = 0.23), also demonstrating zero heterogeneity (I^2^ = 0%, *p* = 0.74).

**Figure 5.**
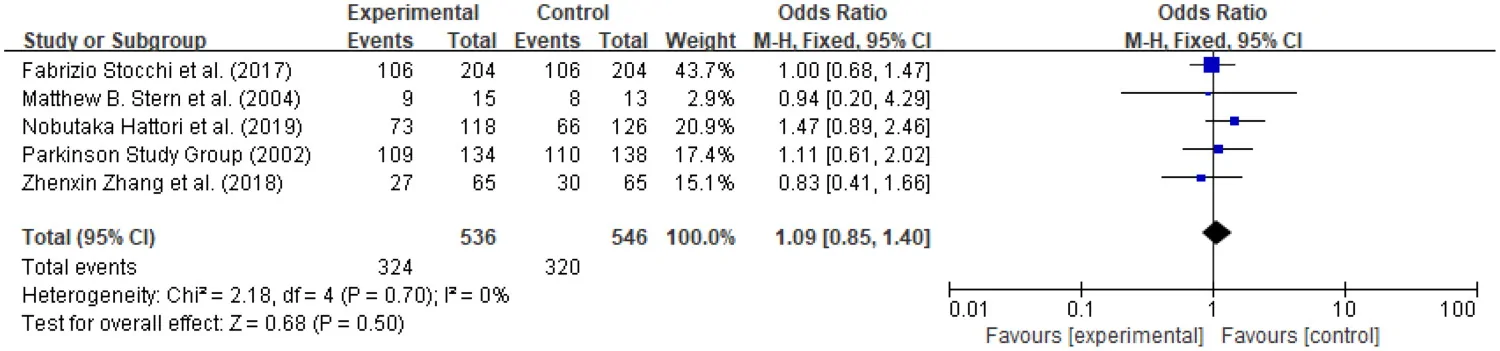
Forest plot of adverse event (AE) incidence comparing rasagiline and placebo. The blue squares represent the point estimate (Odds Ratio) for each individual study, with the size of the square proportional to the study’s weight. The horizontal lines represent the 95% confidence intervals (CIs). The black diamond at the bottom represents the pooled effect size from the direct pairwise meta-analysis. M-H: Mantel-Haenszel method; CI: Confidence Interval; df: degrees of freedom.

**Figure 6.**
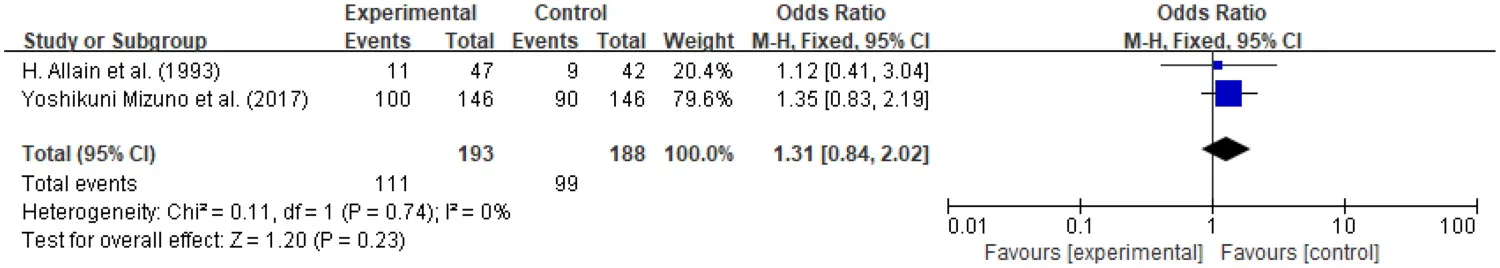
Forest plot of adverse event (AE) incidence comparing selegiline and placebo. The blue squares represent the point estimate (Odds Ratio) for each individual study, with the size of the square proportional to the study’s weight. The horizontal lines represent the 95% confidence intervals (CIs). The black diamond at the bottom represents the pooled effect size from the direct pairwise meta-analysis. M-H: Mantel-Haenszel method; CI: Confidence Interval; df: degrees of freedom.

**Figure 7.**
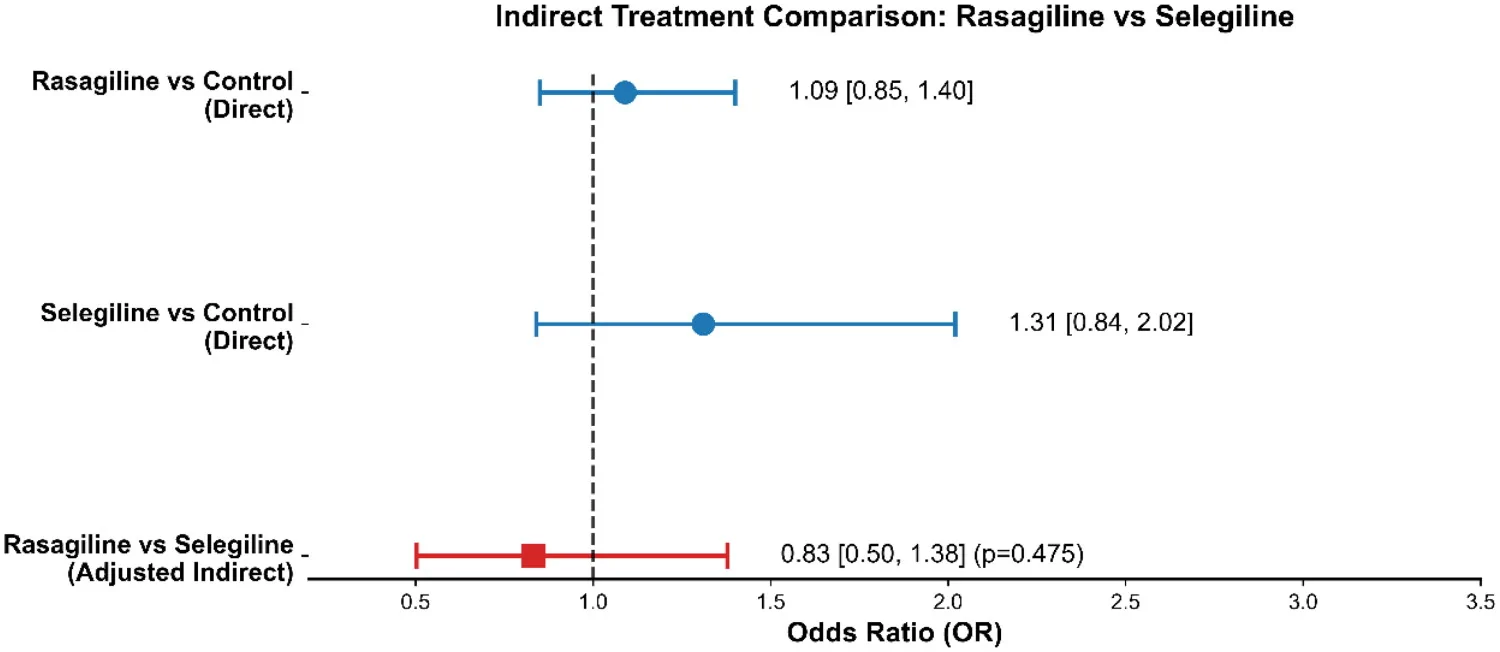
Adjusted indirect treatment comparison (ITC) of adverse event (AE) incidence between rasagiline and selegiline. The blue circles represent the pooled Odds Ratios (ORs) from the direct pairwise meta-analyses of rasagiline or selegiline versus control (placebo). The red square indicates the derived estimate from the Bucher indirect comparison of rasagiline versus selegiline. Horizontal lines denote the 95% confidence intervals (CIs). The vertical dashed line at 1.0 represents the line of no effect. OR: Odds Ratio; ITC: Indirect Treatment Comparison.

To evaluate the comparative safety profile of the two active treatments, an adjusted indirect treatment comparison was performed utilizing the Bucher method [34]. The synthesized indirect evidence revealed no statistically significant difference in the risk of adverse events between rasagiline and selegiline (OR = 0.83, 95% CI [0.50, 1.38], *p* = 0.475). Consequently, both agents exhibit comparable and highly favorable safety profiles when utilized as early-stage monotherapy for Parkinson’s disease. See Figures 5–7.

#### 3.1.3. Subgroup analysis

As pre-specified in our methodology, subgroup analyses were planned to explore potential sources of variance in the event of substantial statistical heterogeneity. However, the direct pairwise meta-analyses revealed absolute statistical homogeneity across the included trials for both efficacy and safety outcomes. Consequently, the predefined criteria for initiating subgroup analyses were not met, and these analyses were deliberately waived. This remarkable consistency across the studies reinforces the stability of our pooled estimates and indicates that our strict inclusion criteria (focusing solely on early-stage monotherapy) successfully minimized clinical and methodological confounding factors.

#### 3.1.4. Sensitivity analysis

**Figure 8.**
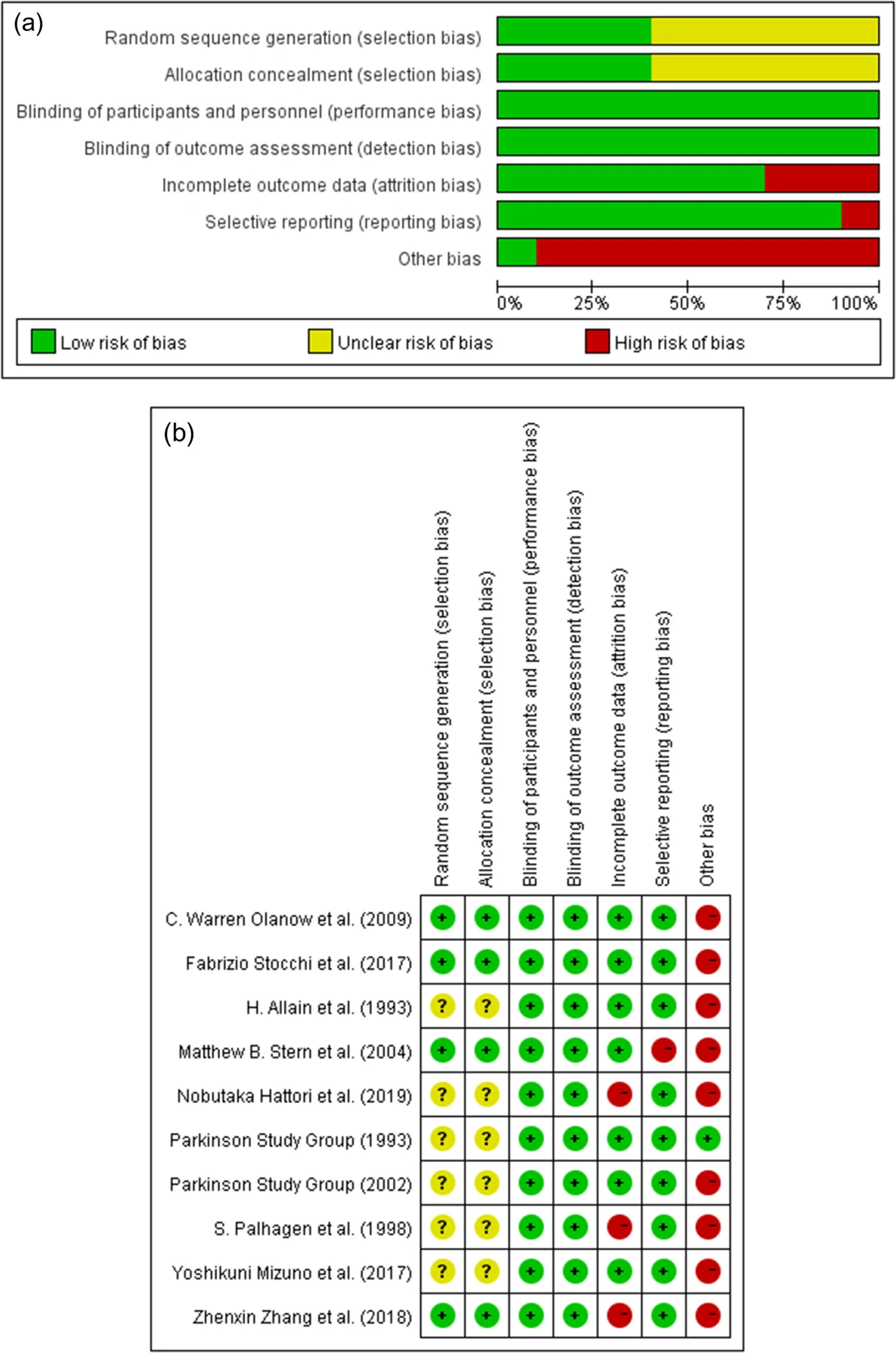
Risk of bias assessment. (a) Risk of bias graph: review authors’ judgements about each risk of bias item presented as percentages across all included studies. (b) Risk of bias summary: review authors’ judgements about each risk of bias item for each included study. Green (+): Low risk of bias; Yellow (?): Unclear risk of bias; Red (−): High risk of bias.

A leave-one-out sensitivity analysis was conducted to assess the robustness of the meta-analysis. The sequential exclusion of each individual study did not yield any substantial changes in the pooled effect estimates or their statistical significance, thereby confirming the high stability and robustness of our overall findings. The quality assessment of each study is shown in Figure 8.

#### 3.1.5. Publication bias

We initially planned to assess potential publication bias and small-study effects by visual inspection of contour-enhanced funnel plots and formal statistical testing (e.g. Egger’s regression test). However, according to the guidelines from the Cochrane Handbook for Systematic Reviews [37] of Interventions, tests for funnel plot asymmetry should only be used when there are at least 10 studies included in the meta-analysis. Because our indirect comparison network comprised a limited number of trials (6 evaluating rasagiline and 4 evaluating selegiline), the statistical power would be inadequate to distinguish chance from real asymmetry. Therefore, formal assessment of publication bias was deemed inappropriate and was not conducted.

#### 3.1.6. GRADE assessment for the certainty of evidence

The certainty of evidence for the indirect comparison between rasagiline and selegiline was assessed using the Grading of Recommendations Assessment, Development and Evaluation (GRADE) [38] approach. All 10 included studies were randomized, double-blind, placebo-controlled trials, yielding an initial evidence rating of high certainty. After a comprehensive evaluation of the five GRADE domains, the overall quality of evidence was rated as moderate.

The evidence was downgraded by one level solely for indirectness (−1). Because there were no head-to-head randomized controlled trials directly comparing rasagiline with selegiline, the relative efficacy was estimated through an indirect comparison network using placebo as the common anchor.

Importantly, the evidence was not downgraded for the following domains:

Inconsistency (0): Although the included trials had varying total follow-up durations in their original designs, our meta-analysis methodologically restricted data extraction to short-term outcomes across all studies. This approach successfully eliminated the clinical heterogeneity related to trial duration, demonstrating consistent positive treatment effects for both drugs in the short term.
Publication Bias (0): A comprehensive and exhaustive literature search was conducted. Given that the majority of the included trials—regardless of their sample sizes—consistently reported positive short-term symptomatic benefits, and considering the highly regulated nature of these pivotal trials, the risk of significant publication bias was deemed low.
Risk of bias and Imprecision (0): Risk of bias was generally low across the well-designed trials, and the cumulative sample size was sufficiently large to provide precise effect estimates.

In conclusion, the moderate certainty of evidence suggests that we can be moderately confident in the effect estimate, although future direct head-to-head trials may still refine our understanding of the comparative efficacy.

## 3.2. Results of pharmacoeconomic research

### 3.2.1. Model structure diagram

**Figure 9.**
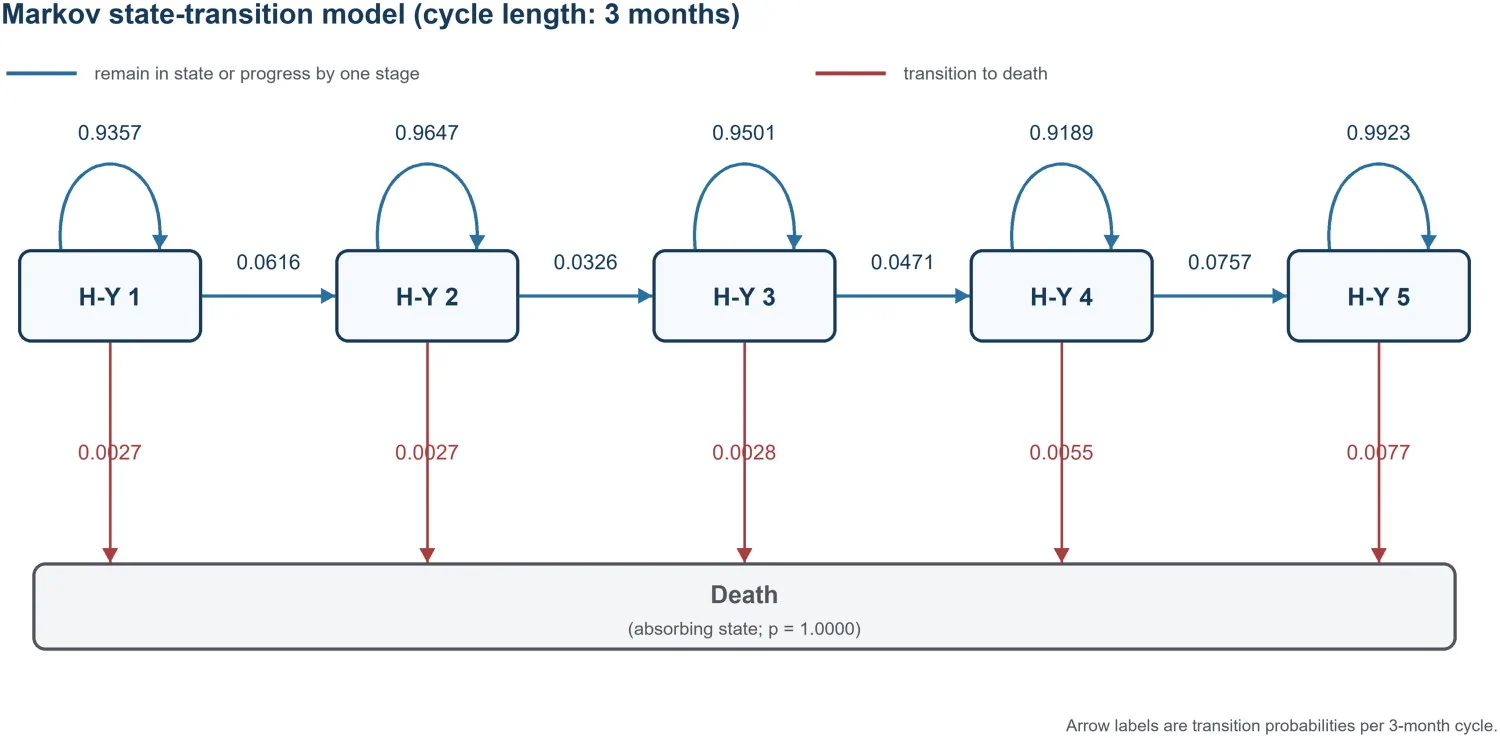
Schematic representation of the Markov model structure. The model simulates the progression of Parkinson’s disease through Hoehn and Yahr (H-Y) stages 1–5 and Death. Patients can remain in the same stage, progress to the next stage, or die. The cycle length is 3 months. H-Y: Hoehn and Yahr stage.

A simplified schematic diagram of the model is shown in Figure 9.

### 3.2.2. Outcome indicators

Treatment cost: Direct drug costs for Rasagiline are 2840.25 ± 932.02 YUAN/3M, and for Selegiline, it is 356.89 ± 30.05 YUAN/3M. Based on expert consultation and a comprehensive review of relevant literature, the cost associated with the management of each adverse event (AE) was assumed to be 200 RMB (To account for uncertainty, this parameter was varied by ± 50% in the deterministic sensitivity analysis), accompanied by a utility decrement of 0.05 quality-adjusted life-years (QALYs) per episode. And the incidence rates come from our meta-analysis. To avoid double-counting, the aforementioned economic and utility decrements were only incorporated during the first simulation cycle (stage 0).

After synthesizing the baseline data with the efficacy outcomes derived from our meta-analysis, the estimated UPDRS scores across each Hoehn and Yahr (H&Y) stage for the two treatment cohorts are presented in Table 2

**Table 2.** Quality of life scores for patients at different stages under selegiline and rasagiline intervention.

	H-Y 1 mean ± SD	H-Y 2 mean ± SD	H-Y 3 mean ± SD	H-Y 4 mean ± SD	H-Y 5 mean ± SD
Selegiline UPDRSII	4.67 ± 7.00	9.15 ± 8.22	14.86 ± 8.70	24.62 ± 8.00	33.54 ± 7.53
Selegiline UPDRSIII	7.54 ± 12.76	20.40 ± 15.36	30.66 ± 16.77	47.33 ± 13.65	58.71 ± 13.74
Rasagiline UPDRSII	4.10 ± 5.82	8.58 ± 7.25	14.29 ± 7.78	24.62 ± 8.00	33.54 ± 7.53
Rasagiline UPDRSIII	7.06 ± 9.07	19.92 ± 12.47	30.18 ± 14.17	47.33 ± 13.65	58.71 ± 13.74

The transition probability matrix, derived using the methodology described above, is presented in Table 3.

**Table 3.** Transition probabilities of disease states in patients with Parkinson’s disease.

	H-Y 1	H-Y 2	H-Y 3	H-Y 4	H-Y 5	Dead
H-Y 1	0.9357	0.0616	0	0	0	0.0027
H-Y 2	0	0.9647	0.0326	0	0	0.0027
H-Y 3	0	0	0.9501	0.0471	0	0.0028
H-Y 4	0	0	0	0.9189	0.0757	0.0055
H-Y 5	0	0	0	0	0.9923	0.0077

In the base-case analysis, treatment with rasagiline yielded a marginal health benefit of 0.0088 QALYs compared to selegiline, but incurred an additional cost of 17,111.10. This resulted in an incremental cost-effectiveness ratio (ICER) of 1,951,505.55 per QALY gained, indicating that rasagiline is not a cost-effective alternative at conventional willingness-to-pay thresholds.

### 3.2.3. Sensitivity analysis

One-way sensitivity analysis: According to the ‘Chinese Guidelines for Pharmacoeconomic Evaluation’ [13], The cost of managing an adverse event was varied by ±50%, whereas the other included parameters were varied by ±10%. The factors significantly influencing the ICER were the utility values of H-Y stages and the discount rate.

**Figure 10.**
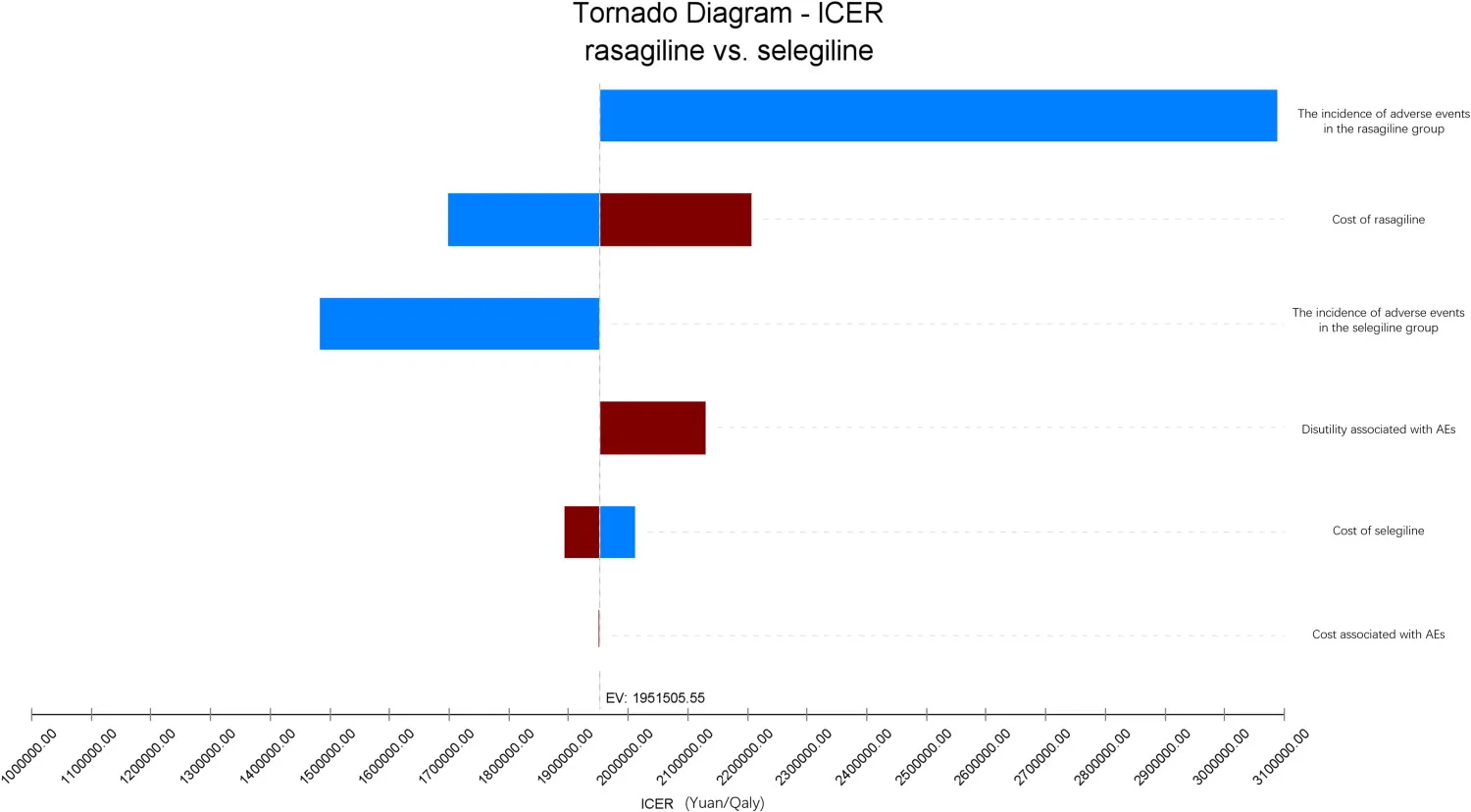
Tornado diagram from one-way sensitivity analysis. The bars indicate the range of Incremental Cost-Effectiveness Ratio (ICER) variations when each parameter is varied within its specified range. ICER: Incremental Cost-Effectiveness Ratio; H-Y: Hoehn and Yahr; WTP: Willingness-to-Pay (287,247 YUAN/QALY).

One-way deterministic sensitivity analyses were conducted to evaluate the robustness of the base-case results. For details, see Figure 10. The ICER was most sensitive to the incidence of adverse events in the rasagiline group, followed by the drug costs of rasagiline and selegiline and the incidence of adverse events in the selegiline group. The disutility and management cost associated with adverse events had relatively small effects. In all tested scenarios, the ICER remained above the prespecified willingness-to-pay threshold. Health-state utility parameters were excluded from the tornado diagram because the small base-case difference in QALYs meant that minor changes in utility could cause incremental effectiveness to cross zero, producing extremely large or undefined ICERs. It is noteworthy that specific health state utility parameters were omitted from the one-way sensitivity analysis. Because the baseline incremental QALY between the evaluated strategies was minimal, slight variations in utility inputs would cause the incremental effectiveness to cross zero, artificially inflating the ICER to infinity. Excluding these variables allowed us to more accurately isolate and present the impact of economic parameters and disease progression rates without visual distortion

#### 3.2.3.1. Probabilistic sensitivity analysis (PSA)

**Figure 11.**
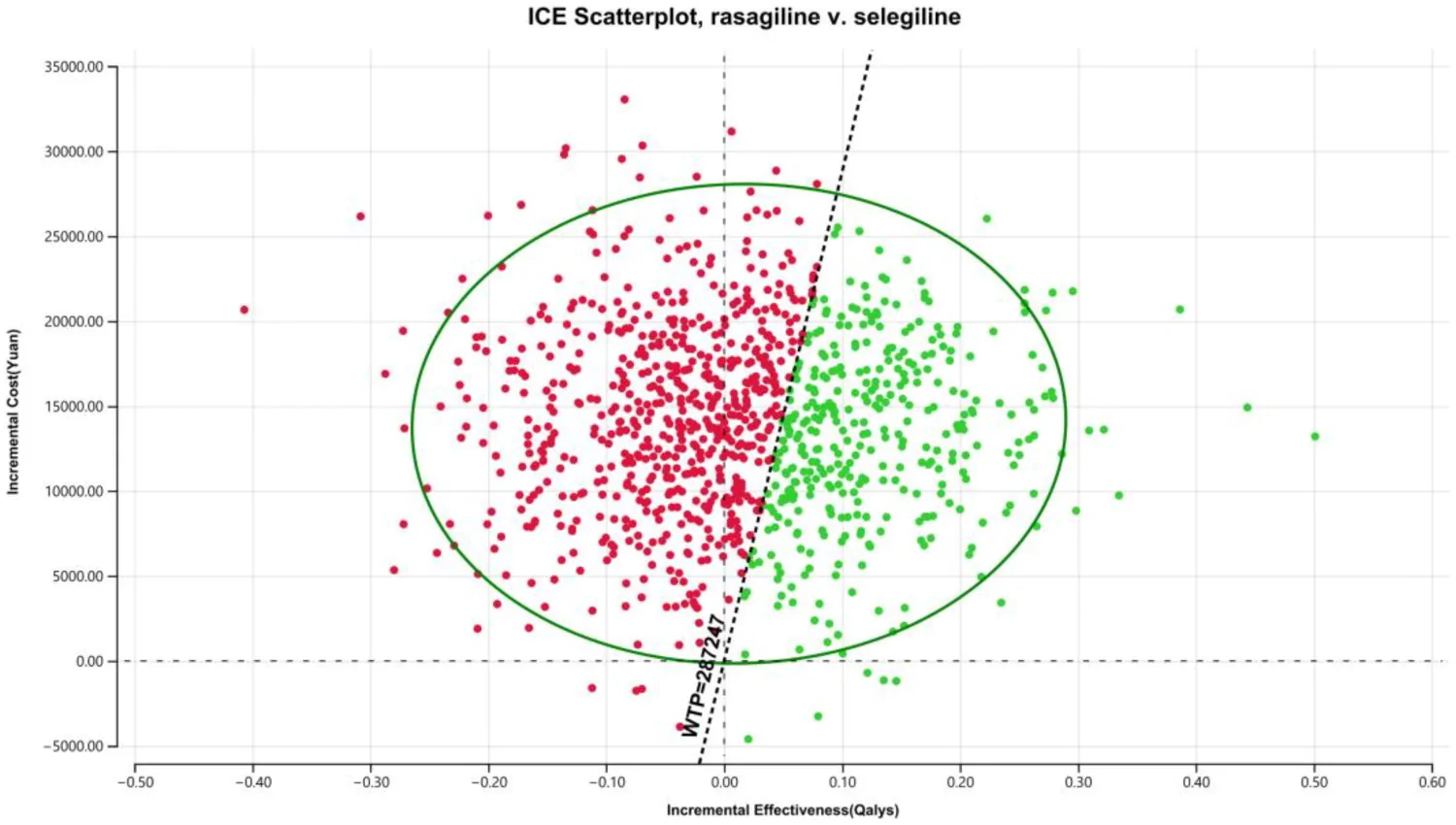
Probabilistic sensitivity analysis scatter plot (10,000 iterations). Each dot represents a simulation result of incremental cost versus incremental effectiveness. The ellipse represents the 95% confidence ellipsoid. Points below the WTP threshold line (dashed line) indicate that Rasagiline is cost-effective. WTP: Willingness-to-Pay (287,247 RMB/QALY); QALY: Quality-Adjusted Life Year; YUAN: Chinese YUAN.

A probabilistic sensitivity analysis using 10,000 Monte Carlo simulations was performed to assess the joint uncertainty of all model parameters simultaneously. The results are depicted in the incremental cost-effectiveness (ICE) scatterplot. See Figure 11. The scatterplot illustrates the distribution of the 10,000 simulated iterations comparing rasagiline against selegiline on the cost-effectiveness plane. The majority of the simulated iterations clustered in the northeast quadrant, indicating that while rasagiline yields slight clinical benefits (incremental effectiveness > 0), it is consistently associated with higher incremental costs. At a defined WTP threshold of 287,247 RMB/QALY (represented by the dashed diagonal line), a substantial proportion of the simulated points fell to the left of the threshold line (red dots). This graphically confirms that at the current pricing, the probability of rasagiline being considered a cost-effective alternative over selegiline is relatively low.

Because the incremental health benefit (QALY) between rasagiline and selegiline was marginal in our base-case analysis, the denominator effect rendered the absolute numerical values of the ICER highly sensitive and statistically unstable in the probabilistic sensitivity analysis (PSA). To align with best practices in health economic evaluation, we avoided reporting the potentially misleading mathematical mean of the simulated ICERs.

Scenario Analysis: Assumption of Equal Efficacy Given the heterogeneity observed in our meta-analysis: lack of significant difference in the efficacy indicator, we conducted an additional scenario analysis assuming equal clinical efficacy for both Rasagiline and Selegiline. Under this conservative assumption, Rasagiline provides no incremental QALY gain but incurs higher costs (Incremental Cost = 17111.1 Yuan). In this scenario, Selegiline strictly dominates Rasagiline, highlighting that the economic value of Rasagiline is entirely dependent on the certainty of its symptomatic superiority.

## 4. Discussion

This study comprehensively assessed the comparative efficacy, safety, and cost-effectiveness of rasagiline versus selegiline. Given the persistent lack of direct head-to-head trials, our findings represent a rigorous quantitative synthesis utilizing an adjusted indirect treatment comparison. While these results should be interpreted within the context of moderate-certainty evidence, they offer the most systematic evaluation of short-term early monotherapy currently available.

### 4.1. Summary of exploratory findings

Our findings, grounded in moderate-certainty evidence, indicate comparable short-term efficacy and safety between rasagiline and selegiline. However, the marginal incremental clinical benefit renders rasagiline not cost-effective at current pricing, necessitating significant price adjustments or long-term clinical superiority to justify its economic value.

### 4.2. Interpretation of divergent efficacy

Our analysis focuses explicitly on the early stages of monotherapy and short-term efficacy, yet these findings prompt an intriguing discussion when contrasted with long-term observational trajectories. While our indirect treatment comparison indicates that both rasagiline and selegiline provide comparable and significant short-term symptomatic relief, historical long-term retrospective data occasionally suggest a sustained functional advantage for selegiline. This divergence between short-term efficacy and long-term subjectively perceived outcomes may be driven by distinct pharmacological mechanisms. Rasagiline, as a potent and irreversible MAO-B inhibitor devoid of amphetamine-derived metabolites, offers a clean safety profile perfectly suited for immediate short-term symptomatic control [39]. Conversely, the metabolism of selegiline yields L-methamphetamine and L-amphetamine. Although these metabolites raise safety concerns, their inherent psychostimulant properties might mask subjective functional decline in long-term observational scenarios, potentially sustaining patient-reported UPDRS scores over extended horizons [40]. Additionally, the notion of delayed disease progression historically linked to selegiline—rooted in enduring debates since the DATATOP trial—provides a plausible context for why long-term retrospective variations exist despite comparable short-term efficacy profiles [41].

### 4.3. Limitations

Several limitations must be acknowledged. First, due to the persistent lack of head-to-head RCTs, our findings rely entirely on an adjusted indirect treatment comparison. Consequently, despite effectively minimizing inter-study heterogeneity, the overall certainty of evidence was downgraded to ‘Moderate’ according to the GRADE framework. Second, our meta-analysis intentionally restricted evaluations to short-term outcomes (10–16 weeks) to optimize homogeneity. While methodologically robust, this precludes drawing definitive conclusions regarding the long-term sustainability or potential disease-modifying effects of these treatments. Third, the pharmacoeconomic evaluation utilized transition probabilities and baseline clinical parameters derived from international cohorts, which may imperfectly reflect the epidemiological characteristics of the Chinese population. Finally, our economic model inherently omitted non-medical costs and variations in real-world patient adherence, which might underestimate the total economic complexity of lifelong PD management.

In the future, multi-center, large-sample prospective cohort studies can be conducted for the Chinese PD patient population, focusing on collecting medical resource data on long-term medication, adherence, and management of non-motor symptoms. By combining electronic health records (EHR) and medical insurance databases, the actual efficacy differences of Rasagiline in patients at different stages can be analyzed, providing dynamic calibration for model parameters.

## 5. Conclusion

Current adjusted indirect evidence suggests that rasagiline and selegiline offer comparable short-term symptomatic relief and safety profiles for early-stage Parkinson’s disease. However, rasagiline is currently not a cost-effective alternative due to its substantially higher pricing. Until significant price reductions are implemented or robust head-to-head trials definitively prove its long-term clinical superiority, selegiline remains the more economically viable option for initial monotherapy.

## Data Availability

Authors agree to make data and materials supporting the results or analyses presented in their paper available upon reasonable request.

## Protocol and registration

This systematic review and meta-analysis were designed and conducted in accordance with the Preferred Reporting Items for Systematic Reviews and Meta-Analyses (PRISMA) 2020 guidelines. The protocol was prospectively registered with the International Prospective Register of Systematic Reviews (PROSPERO). Available from https://www.crd.york.ac.uk/PROSPERO/view/CRD420251153722. The registration number is CRD420251153722. The protocol is available from https://www.crd.york.ac.uk/PROSPEROFILES/9d91ca134a99cd216f6ccaf044bd9b37.pdf.

1 minor amendment and 1 major amendment was made to the registered protocol during the conduct of this review.

## References

[1] Xu T, Dong W, Liu J, et al. Disease burden of Parkinson’s disease in China and its provinces from 1990 to 2021: findings from the global burden of disease study 2021. Lancet Reg Health West Pac. 2024;46:101078. doi: 10.1016/j.lanwpc.2024.101078. 38745974 PMC11091691

[2] Xu J, Gong DD, Man CF, et al. Parkinson’s disease and risk of mortality: meta-analysis and systematic review. Acta Neurol Scand. 2014;129(2):71–79. doi: 10.1111/ane.12201. 24256347

[3] Liu T, Wang Y, , et al. Investigation on the economic burden of Parkinson ‘ s disease and its influencing factors. J Clin Neurol. 2022;35(3):166–170.Chinese

[4] Yan R, Cai H, Cui Y, et al. Comparative efficacy and safety of monoamine oxidase type B inhibitors plus channel blockers and monoamine oxidase type B inhibitors as adjuvant therapy to levodopa in the treatment of Parkinson’s disease: a network meta-analysis of randomized controlled trials. Eur J Neurol. 2023;30(4):1118–1134. doi: 10.1111/ene.15651. 36437702

[5] Binde CD, Tvete IF, Gåsemyr JI, et al. Comparative effectiveness of dopamine agonists and monoamine oxidase type-B inhibitors for Parkinson’s disease: a multiple treatment comparison meta-analysis. Eur J Clin Pharmacol. 2020;76(12):1731–1743. doi: 10.1007/s00228-020-02961-6. 32710141 PMC7661406

[6] Wang Y, Wang Z. Effects and safety of monoamine oxidase-B inhibitors for early Parkinson’s disease: a network meta-analysis. Eur Neurol. 2024;87(5-6):273–290. doi: 10.1159/000541315. 39278214

[7] Binde CD, Tvete IF, Gåsemyr J, et al. A multiple treatment comparison meta-analysis of monoamine oxidase type B inhibitors for Parkinson’s disease. Br J Clin Pharmacol. 2018;84(9):1917–1927. doi: 10.1111/bcp.13651. 29847694 PMC6089809

[8] Huang YH, Chen JH, Loh EW, et al. The effect of monoamine oxidase-B inhibitors on the alleviation of depressive symptoms in Parkinson’s disease: meta-analysis of randomized controlled trials. Ther Adv Psychopharmacol. 2021;11:2045125320985993. doi: 10.1177/2045125320985993. 33520156 PMC7816524

[9] Siddiqui MA, Plosker GL. Rasagiline. Drugs Aging. 2005;22(1):83–91. doi: 10.2165/00002512-200522010-00006. 15663351

[10] Chen JJ, Swope DM, Dashtipour K. Comprehensive review of rasagiline, a second-generation monoamine oxidase inhibitor, for the treatment of Parkinson’s disease. Clin Ther. 2007;29(9):1825–1849. doi: 10.1016/j.clinthera.2007.09.021. 18035186

[11] Connolly BS, Lang AE. Pharmacological treatment of Parkinson disease: a review. JAMA. 2014;311(16):1670‐–1683. doi: 10.1001/jama.2014.3654. 24756517

[12] Husereau D, Drummond M, Augustovski F, et al. Consolidated health economic evaluation reporting standards (CHEERS) 2022 explanation and elaboration: a report of the ISPOR CHEERS II good practices task force. Value Health. 2022;25(1):10–31. doi: 10.1016/j.jval.2021.10.008. 35031088

[13] Liu G, Hu S, Wu J. China guidelines for pharmacoeconomic evaluations. China J Pharma Econ. 2011;3:6–48.

[14] Cai D, Shi S, Jiang S, et al. Estimation of the cost-effective threshold of a quality-adjusted life year in China based on the value of statistical life. Eur J Health Econ. 2022;23(4):607–615. doi: 10.1007/s10198-021-01384-z. 34655364 PMC9135816

[15] Martinez-Martin P, Skorvanek M, Rojo-Abuin JM, et al. Validation study of the hoehn and yahr scale included in the MDS-UPDRS. Mov Disord. 2018;33(4):651–652. doi: 10.1002/mds.27242. 29322548

[16] Johnson SJ, Diener MD, Kaltenboeck A, et al. An economic model of Parkinson’s disease: implications for slowing progression in the United States. Mov Disord. 2013;28(3):319–326. 23404374 10.1002/mds.25328

[17] National Bureau of Statistics of China. China Population Census Yearbook 2020. Beijing: China Statistics Press; 2022.

[18] Goetz CG, Stebbins GT, Tilley BC. Calibration of unified Parkinson’s disease rating scale scores to Movement Disorder Society-unified Parkinson’s disease rating scale scores. Mov Disord. 2012;27(10):1239–1242. doi: 10.1002/mds.25122. 22886777

[19] Hoehn MM, Yahr MD. Parkinsonism: onset, progression and mortality. Neurology. 1967;17(5):427–442. doi: 10.1212/wnl.17.5.427. 6067254

[20] Goetz CG, Poewe W, Rascol O, et al. Movement Disorder Society Task Force report on the Hoehn and Yahr staging scale: status and recommendations. Mov Disord. 2004;19(9):1020–1028. doi: 10.1002/mds.20213. 15372591

[21] Dams J, Klotsche J, Bornschein B, et al. Mapping the EQ-5D index by UPDRS and PDQ-8 in patients with Parkinson’s disease. Health Qual Life Outcomes. 2013;11(1):35. doi: 10.1186/1477-7525-11-35. 23497005 PMC3662160

[22] Stocchi F, Rascol O, Hauser RA, et al. Randomized trial of preladenant, given as monotherapy, in patients with early Parkinson disease. Neurology. 2017;88(23):2198–2206. doi: 10.1212/WNL.0000000000004003. 28490648

[23] Stern MB, Marek KL, Friedman J, et al. Double-blind, randomized, controlled trial of rasagiline as monotherapy in early Parkinson’s disease patients. Mov Disord. 2004;19(8):916–923. 15300656 10.1002/mds.20145

[24] Olanow CW, Rascol O, Hauser R, et al. A double-blind, delayed-start trial of rasagiline in Parkinson’s disease. N Engl J Med. 2009;361(13):1268–1278. doi: 10.1056/NEJMoa0809335. 19776408

[25] Parkinson Study Group. A controlled trial of rasagiline in early Parkinson disease: the TEMPO study. Arch Neurol. 2002;59(12):1937–1943. 12470183 10.1001/archneur.59.12.1937

[26] Hattori N, Takeda A, Takeda S, et al. Rasagiline monotherapy in early Parkinson’s disease: a phase 3, randomized study in Japan. Parkinsonism Relat Disord. 2019;60:146–152. doi: 10.1016/j.parkreldis.2018.08.024. 30205936

[27] Zhang Z, Wang J, Chen S, et al. Efficacy and safety of rasagiline in Chinese patients with early Parkinson’s disease: a randomized, double-blind, parallel, placebo-controlled, fixed-dose study. Transl Neurodegener. 2018;7:32. doi: 10.1186/s40035-018-0137-5. 30534374 PMC6282325

[28] Pålhagen S, Heinonen E, Hägglund J, et al. Selegiline slows the progression of the symptoms of Parkinson disease. Neurology. 2006;66(8):1200–1206. doi: 10.1212/01.wnl.0000204007.46190.54. 16540603

[29] Mizuno Y, Hattori N, Kondo T, et al. A Randomized Double-Blind Placebo-Controlled Phase III Trial of Selegiline Monotherapy for Early Parkinson Disease. Clin Neuropharmacol. 2017;40(5):201–207. 28857772 10.1097/WNF.0000000000000239 PMC5610558

[30] Allain H, Pollak P, Neukirch HC. Symptomatic effect of selegiline in de novo Parkinsonian patients. The French Selegiline Multicenter Trial. Mov Disord. 1993;8(Suppl 1):S36–S40. 8302306 10.1002/mds.870080508

[31] Parkinson Study Group. Effects of tocopherol and deprenyl on the progression of disability in early Parkinson’s disease. N Engl J Med. 1993;328(3):176–183. doi: 10.1056/NEJM199301213280305. 8417384

[32] Page MJ, Mckenzie JE, Bossuyt PM, et al. The PRISMA 2020 statement: an updated guideline for reporting systematic reviews. BMJ. 2021;372:n71. 10.1136/bmj.n71 33782057 33782057 PMC8005924

[33] Page MJ, Moher D, Bossuyt PM, et al. PRISMA 2020 explanation and elaboration: updated guidance and exemplars for reporting systematic reviews. BMJ. 2021;372:n160. 10.1136/bmj.n160 33781993 33781993 PMC8005925

[34] Bucher HC, Guyatt GH, Griffith LE, et al. The results of direct and indirect treatment comparisons in meta-analysis of randomized controlled trials. J Clin Epidemiol. 1997;50(6):683–691. 10.1016/s0895-4356(97)00049-8 9250266 9250266

[35] Peretz C, Segev H, Rozani V, et al. Comparison of selegiline and rasagiline therapies in parkinson disease: a real-life study. Clin Neuropharmacol. 2016;39(5):227–231. doi: 10.1097/WNF.0000000000000167. 27438181 PMC5028154

[36] Cereda E, Cilia R, Canesi M, et al. Efficacy of rasagiline and selegiline in Parkinson’s disease: a head-to-head 3-year retrospective case-control study. J Neurol. 2017 Jun;264(6):1254-1263. doi: 10.1007/s00415-017-8523-y PMC5570795 28550482

[37] Higgins JPT, Thomas J, Chandler J, Cumpston M, Li T, Page MJ, et al, editor(s). Cochrane Handbook for Systematic Reviews of Interventions version 6.5 (updated August 2024). Cochrane, 2024. Available from https://www.cochrane.org/handbook.

[38] Guyatt GH, Oxman AD, Vist GE, et al. GRADE: an emerging consensus on rating quality of evidence and strength of recommendations. BMJ. 2008;336(7650):924–926. 10.1136/bmj.39489.470347.AD 18436948 18436948 PMC2335261

[39] Chen JJ, Swope DM. Clinical pharmacology of rasagiline: a novel, second-generation propargylamine for the treatment of Parkinson disease. J Clin Pharmacol. 2005;45(8):878–894. doi: 10.1177/0091270005277935. 16027398

[40] Chrisp P, Mammen GJ, Sorkin EM. Selegiline. A review of its pharmacology, symptomatic benefits and protective potential in Parkinson’s disease. Drugs Aging. 1991;1(3):228–248. doi: 10.2165/00002512-199101030-00006. 1794016

[41] Parkinson Study Group. Effect of deprenyl on the progression of disability in early Parkinson’s disease. N Engl J Med. 1989;321(20):1364–1371. 2509910 10.1056/NEJM198911163212004

